# State Prior Authorization Prohibitions and Buprenorphine Retention Among Privately Insured Patients

**DOI:** 10.1001/jamahealthforum.2026.0012

**Published:** 2026-03-06

**Authors:** Ju-Chen Hu, Shashi N. Kapadia, Hao Zhang, Ali Jalali, Kristen Underhill, Christina M. Andrews, Yuhua Bao

**Affiliations:** 1 Department of Health Policy and Management, Celia Scott Weatherhead School of Public Health and Tropical Medicine, Tulane University, New Orleans, Louisiana; 2 Department of Population Health Sciences, Weill Cornell Medicine, New York, New York; 3 Division of Infectious Diseases, Weill Cornell Medicine, New York, New York; 4 Department of Health Policy and Organization, School of Public Health, University of Alabama at Birmingham, Birmingham; 5 Cornell Law School, Ithaca, New York; 6 Department of Health Services Policy and Management, Arnold School of Public Health, University of South Carolina, Columbia

## Abstract

**Importance:**

Buprenorphine is the most commonly prescribed medication for opioid use disorder, but rates of retention in treatment remain low. Prior authorization (PA) has been identified as a barrier to the administration of buprenorphine, and patients may need multiple PAs to continue treatment; thus, many states have passed laws to prohibit the use of PA for buprenorphine in private insurance.

**Objective:**

To examine the association between PA prohibitions and buprenorphine treatment retention overall and by branded vs generic drug status.

**Design, Setting, and Participants:**

This cross-sectional study used a difference-in-differences design and private insurance claims data from 49 states and the District of Columbia with no PA prohibition for buprenorphine as of January 1, 2015. Patients aged 18 to 64 years who started a new buprenorphine treatment between January 1, 2015, and June 1, 2022, were included in the study. Data were analyzed from June 3, 2024, to December 31, 2025.

**Exposure:**

Prior authorization prohibitions, defined as state laws barring private insurers from using PA for any buprenorphine product.

**Main Outcomes and Measures:**

The main outcome was buprenorphine treatment retention, measured by a dichotomous variable indicating whether the buprenorphine treatment episode lasted 180 days or longer.

**Results:**

The sample included 22 946 patients (67.7% male) who started buprenorphine treatment; 54.3% started buprenorphine treatment with generic buprenorphine. A total of 30.4% of patients reached the 180-day treatment retention threshold. Adopting PA prohibitions was not associated with significant changes in buprenorphine treatment retention (effect estimate, 0.007; 95% CI, −0.044 to 0.059; *P* = .78). Stratified analysis by branded vs generic status of buprenorphine at the start of the treatment episode showed no significant association between PA prohibitions and treatment retention among patients receiving either branded (effect estimate, −0.018; 95% CI, −0.075 to 0.040; *P* = .55) or generic (effect estimate, 0.041; 95% CI, −0.036 to 0.118; *P* = .30) buprenorphine.

**Conclusions and Relevance:**

This cross-sectional study found that state laws prohibiting PA for buprenorphine were not associated with significant changes in buprenorphine treatment retention among privately insured patients. Prior authorization prohibition alone may not be effective in improving buprenorphine treatment retention. Additional interventions appear to be needed to address gaps in opioid use disorder treatment for privately insured patients.

## Key Points

**Question:**

Are state legislative prior authorization prohibitions associated with changes in buprenorphine treatment retention among privately insured patients?

**Findings:**

This cross-sectional study of 22 946 privately insured patients who started buprenorphine treatment from 2015 to 2022 found that state prior authorization prohibitions were not associated with significant changes in whether patients continued treatment for 180 days or longer.

**Meaning:**

The findings suggest that additional interventions are needed to address gaps in opioid use disorder treatment among privately insured patients.

## Teaser

This cross-sectional study examines the association between state laws that prohibit prior authorization for buprenorphine and treatment retention among privately insured patients.

## Introduction

The US continues to face an opioid overdose crisis. While 2023 marked the first year with a decline in opioid-involved deaths since 2018, the number of such deaths still exceeded 100 000 that year.^1^ This death toll persisted despite the availability of effective treatment such as medications for opioid use disorder (MOUD). In prior studies, about 80% of adults with OUD did not receive MOUD,^2,3^ highlighting a substantial gap in OUD treatment.

Buprenorphine is the most widely prescribed MOUD in the US,^4^ but treatment retention remains low. To ensure effective treatment outcomes, patients must continue receiving buprenorphine for a sufficient duration.^5^ However, recent research estimated that only 22% to 47% of patients who initiated buprenorphine treatment reached 180-day retention,^6,7^ a threshold representing the minimum recommended duration of MOUD treatment endorsed by the federal government.^8^ In addition, discontinuing buprenorphine treatment during this period was associated with a higher risk of relapse of opioid use, overdose, and death.^9,10,11,12^ Consequently, ensuring sustained buprenorphine retention for patients initiating treatment for OUD is a critical public health objective.

Prior authorization (PA) is an administrative process that requires health care practitioners to obtain insurer approval for coverage before they deliver certain services or medications. Prior authorization requirements for the same medications can change over time. Consequently, even if patients initiate treatment without a PA, they may still need PAs for refills or when switching to a different product. Moreover, even when PA is approved, the approval is often limited to certain time frames or prescriptions with certain days of supply.^13^ For patients with OUD, PA may hinder treatment retention, as prescribers may need to repeatedly address PAs throughout the treatment course, increasing the risk of interruptions or delays in treatment. Research has also linked PA with reduced practitioner willingness to prescribe,^14,15^ shortened treatment duration,^16^ and delayed treatment.^17,18^ Despite these potential harms of PA, health care practitioners commonly encounter PA requirements. In 2024, physicians and their staff spent an average of 13 hours each week completing requirements for PA, and 94% of physicians reported that PA caused negative impacts on patient clinical outcomes.^19^

About one-third of patients with OUD were covered by private insurance in 2023 (our estimate using the 2023 National Survey on Drug Use and Health^20^). Many states have passed laws prohibiting PA for MOUD to reduce barriers to OUD treatment. These laws can be particularly beneficial for private insurance enrollees, as they are more likely to encounter PA requirements and denials than those enrolled in Medicare or Medicaid.^21,22^ A recent study showed that the number of states with PA prohibition in private insurance increased from 2 in 2015 to 22 in 2023.^23^ Despite this positive policy development, to our knowledge, no studies have examined the impacts of PA prohibitions for MOUD for privately insured patients. Additionally, previous studies on PA prohibition in Medicare and Medicaid used state-level or plan-level aggregate data to examine buprenorphine use,^24,25,26^ limiting the ability to observe patient-level outcomes, such as treatment retention. In this study, we used national private insurance claims data to conduct an evaluation of the association between PA prohibitions and buprenorphine treatment retention among privately insured patients.

## Methods

This cross-sectional study followed the Strengthening the Reporting of Observational Studies in Epidemiology (STROBE) reporting guideline. The study protocol was approved by the Weill Cornell Medicine institutional review board. Informed consent was waived because this study used de-identified data and involved no more than minimal risk.

### State Legislative PA Prohibition

The policy of interest was state legislative PA prohibitions, defined as state laws that prohibit private insurance plans from requiring PA for any buprenorphine product. We included PA prohibitions that took effect at any time from January 1, 2015, to June 1, 2022. During the study period, 19 states newly implemented PA prohibitions for buprenorphine in private insurance (eAppendix 1 in Supplement 1). We excluded Rhode Island because its PA prohibition took effect before 2015. Other states did not implement any PA prohibitions for buprenorphine during the study period. More details on PA prohibitions can be found elsewhere.^23^

### Data and Sample

We used the Health Care Cost Institute private insurance claims database, which covers about one-third of privately insured individuals in the US. Our study sample included privately insured patients aged 18 to 64 years who started a new buprenorphine treatment between January 1, 2015, and June 1, 2022. We focused on enrollees’ first observed buprenorphine treatment to mitigate potential confounding effects related to patients’ treatment history and their past experiences navigating the PA process.

To construct the sample, we used pharmacy claims to identify each enrollee’s first observed buprenorphine prescription. The list of national drug codes for buprenorphine was compiled from Athena,^27^ Micromedex RED BOOK,^28^ the US Food and Drug Administration National Drug Code Directory,^29^ RxNorm files,^30^ and Medicaid and CHIP Business Information Solutions.^31^ Buprenorphine formulations approved exclusively for pain management were excluded.^32^ Because OUD diagnosis is underreported in private insurance claims^33^ and because *International Classification of Diseases, Ninth Revision* diagnosis codes of OUD may be inaccurately reported in claims data,^34^ we did not require patients to have an OUD diagnosis. Using the month of the first observed buprenorphine prescription as the index month, we established a 6-month look-back period before the index month and required patients to have had continuous enrollment during this period. We further required patients to have had continuous enrollment during the index month and the following 6 consecutive months. Because state legislative PA prohibitions do not apply to employer self-funded plans under the Employee Retirement Income Security Act, we excluded enrollees of employer self-funded plans. We provide the sample derivation flowchart in eAppendix 2 in Supplement 1.

### Outcomes

Buprenorphine treatment retention was measured by a dichotomous outcome indicating whether the patient’s buprenorphine treatment episode lasted for at least 180 days, a threshold representing the minimum recommended duration of treatment.^8^ We used the prescription fill date and the number of days of supply from pharmacy claims to determine the days on which patients had buprenorphine in their possession. For early refills, we carried the supply of overlapping days forward to the end of the next filled buprenorphine prescription.^33^ Details of how we constructed buprenorphine treatment episodes are provided in eAppendix 3 in Supplement 1.

### Statistical Analysis

We applied a difference-in-differences (DID) design to examine the association between PA prohibition and buprenorphine treatment retention. Notably, PA prohibitions were adopted at different times across study states, and the effects of PA prohibitions may vary by state and the time of adoption. Because recent studies showed that 2-way fixed effects (TWFE) DID models can cause biased estimates in staggered implementation with potential heterogeneous treatment effects,^35,36^ we used the DID model developed by Callaway and Sant’Anna (CSDID)^36^ to provide unbiased estimations in our main analysis. This method allows the use of either never-treated units (ie, patients in states with no PA prohibitions during the entire study period) or not-yet-treated units (ie, patients in states with no PA prohibitions and those during the pre–policy implementation period in states with eventual PA prohibitions). We presented the results of both reference groups. Results of TWFE models are provided in eAppendix 6 in Supplement 1.

The DID design relies on an assumption that absent the policy intervention (PA prohibitions), the outcome would have followed similar trends between states with and without the policy exposure (ie, the parallel trends assumption). We assessed this assumption and presented the results in eAppendix 4 in Supplement 1.

All models controlled for sex, age, and baseline comorbid conditions associated with substance use during the 6-month look-back period (back pain, neck pain, arthritis and joint pain, other pain, mental health conditions, alcohol use disorder, drug use disorder [including opioids, cocaine, and other drugs; *International Statistical Classification of Diseases and Related Health Problems, Tenth Revision* diagnosis codes F11-F16 and F18-F19], nicotine dependence, and cancer) and the generic vs branded status of the first buprenorphine prescription. Patients with any missing data were excluded from analysis. Data on race and ethnicity were unavailable in the Health Care Cost Institute database.

For a secondary analysis, we conducted a stratified analysis to assess whether the association between PA prohibition and buprenorphine treatment retention differed among patients who started treatment with branded buprenorphine formulations vs generics. Specifically, during the study period, both branded and generic buprenorphine products were available for OUD treatment. A recent study found that in 2021, a higher proportion of private insurance formularies covered generic products without PA compared with their branded counterparts.^37^ Because branded drugs were more likely to be subject to PA, we assessed whether patients who took branded drugs would experience greater effects of PA prohibitions.

For buprenorphine treatment retention, we followed the Centers for Medicare & Medicaid Services (CMS) measure of continuity of pharmacotherapy for OUD^8^ and defined that any gap of more than 7 days without medication ended a treatment episode. To assess the robustness of the findings, we conducted a sensitivity analysis by allowing gaps of up to 14 and 30 days. We also assessed 2 additional retention thresholds (30 and 60 days) for sensitivity analysis.

Analyses were performed using SAS, version 9.4 (SAS Institute Inc) and Stata MP, version 19 (StataCorp LLC). We used the csdid package in Stata for analysis and estimated robust standard errors clustered at the state level. Two-sided *P* < .05 was considered significant. Data were analyzed from June 3, 2024, to December 31, 2025.

## Results

### Sample Characteristics and Unadjusted Outcomes

Of 23 565 patients, 2.6% were excluded from analysis because of missing data. The final analytic sample included 22 946 patients (32.2% female; 67.7% male) who started a new buprenorphine treatment episode between January 1, 2015, and June 1, 2022 (Table). More than two-fifths of these patients (43.7%) were 18 to 34 years of age. Many patients had pain-related conditions: 23.7% had back pain, 18.0% had arthritis and joint pain, and 21.1% had other pain. Mental health comorbidity was also common (41.7%). Notably, fewer than half of the patients (47.3%) had a drug use disorder diagnosis during the 6-month look-back period before they started buprenorphine treatment. Most patients (54.3%) started their treatment with a generic formulation.

**Table.** Characteristics and Unadjusted Outcomes Among Patients Who Started Treatment With Buprenorphine

Characteristic	Patients, No. (%) (N = 22 946)
Sex	
Female	7417 (32.3)
Male	15 529 (67.7)
Age, y	
18-34	10 023 (43.7)
35-44	6385 (27.8)
45-64	6538 (28.5)
Comorbidity during 6-mo look-back period	
Back pain	5436 (23.7)
Neck pain	2597 (11.3)
Arthritis and joint pain	4119 (18.0)
Other pain	4845 (21.1)
Mental health conditions	9560 (41.7)
Alcohol use disorder	2259 (9.8)
Drug use disorder	11 255 (49.0)
Nicotine dependence	2448 (10.7)
Cancer	1131 (4.9)
First buprenorphine prescription was generic	12 785 (55.7)
180-d Retention	
Allowing 7-d gaps	6985 (30.4)
Allowing 14-d gaps	8638 (37.6)
Allowing 30-d gaps	10 494 (45.7)

Less than one-third of the patients (30.4%) continued treatment for at least 180 days without gaps longer than 7 days. The 180-day retention rate remained low even when allowing longer gaps between prescriptions. Less than half of the sample (45.7%) continued treatment without gaps longer than 30 days.

### PA Prohibition and Buprenorphine Retention

**Figure 1.**
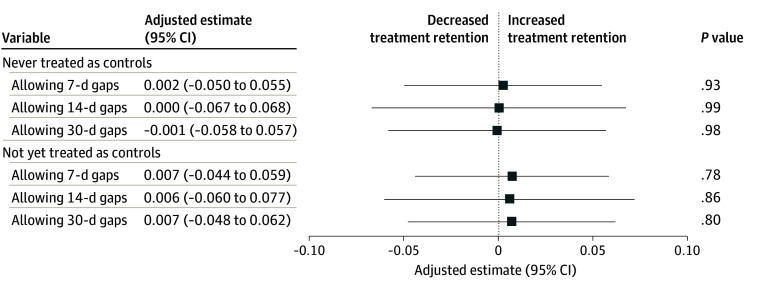
Forest Plot Showing Difference-in-Differences Analysis of the Association Between Prior Authorization Prohibitions and 180-Day Buprenorphine Treatment Retention Patients in states with no prior authorization prohibitions during the entire study period composed the never-treated-as-controls group. Patients in states with no prior authorization prohibitions and those during the pre–policy implementation period in states with eventual prior authorization prohibitions composed the not-yet-treated-as-controls group.

Adopting PA prohibition was not associated with significant changes in 180-day buprenorphine treatment retention. Specifically, PA prohibitions were not associated with a difference in the 180-day treatment retention rate (never treated as controls: estimate, 0.002 [95% CI, −0.050 to 0.055]; *P* = .93; not yet treated as controls: estimate, 0.007 [95% CI, −0.044 to 0.059]; *P* = .78) (Figure 1 and eAppendix 5 in Supplement 1). Analyses of allowing 14-day and 30-day gaps between buprenorphine prescriptions and TWFE models showed consistent results between these 2 gaps (14-day and 30-day), and were also consistent with the main finding (7-day gap) (Figure 1 and eAppendices 5 and 6 in Supplement 1). When lowering the retention threshold to 30 and 60 days, we found that while PA prohibitions were not associated with significant changes in 30-day retention, they were associated with increased 60-day retention (not yet treated as controls, allowing 14-day gaps: estimate, 0.054; 95% CI, 0.004-0.105; *P* = .03) (eAppendix 7 in Supplement 1).

**Figure 2.**
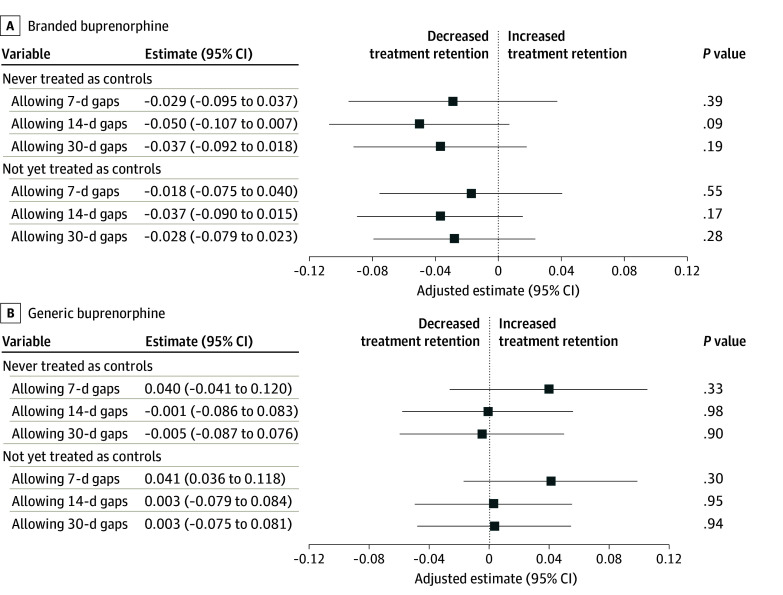
Forest Plots Showing Stratified Analysis of the Association Between Prior Authorization Prohibitions and 180-Day Buprenorphine Treatment Retention by Branded vs Generic Status Patients in states with no prior authorization prohibitions during the entire study period composed the never-treated-as-controls group. Patients in states with no prior authorization prohibitions and those during the pre–policy implementation period in states with eventual prior authorization prohibitions composed the not-yet-treated-as-controls group.

When stratifying by the generic vs branded status of the first buprenorphine prescription, we observed similar results (Figure 2). Stratified analysis by branded vs generic status of buprenorphine at the start of the treatment episode showed no significant association between PA prohibitions and treatment retention among patients receiving either branded (effect estimate, −0.018; 95% CI, −0.075 to 0.040; *P* = .55) or generic (effect estimate, 0.041; 95% CI, −0.036 to 0.118; *P* = .30) buprenorphine. While the TWFE models showed that PA prohibitions were associated with a significant increase in 180-day retention among patients who initiated branded buprenorphine when allowing 7-day and 14-day gaps (eAppendix 6 in Supplement 1), our main analysis using CSDID models showed no associations (Figure 2 and eAppendix 5 in Supplement 1). Among patients who initiated treatment with generic products, PA prohibitions were not associated with significant changes in 180-day retention in the CSDID or TWFE model.

## Discussion

In this study, we found that state legislative PA prohibitions in private insurance were not associated with significant changes in 180-day buprenorphine treatment retention. We also found that PA prohibitions were not associated with buprenorphine treatment retention among patients initiating either a branded or generic version. As more states enact similar PA prohibitions to combat the ongoing opioid epidemic, our findings provide timely and important implications to inform future interventions.

Our findings that adopting PA prohibitions was not associated with significant changes in buprenorphine treatment retention among privately insured patients align with those of studies of buprenorphine use among Medicaid patients. Two studies on Medicaid found that removing PA requirements for buprenorphine was not associated with significant changes in state-level use of buprenorphine prescriptions (except in Illinois).^24,25^ However, studies of Medicare patients showed different findings. Specifically, the percentage of plans requiring PA decreased from 87.51% in 2017 to 3.45% in 2019 for branded buprenorphine and from 95.80% to 0.09% for generic buprenorphine after the CMS announced in 2018 that Medicare Part D formularies requiring more than 1 PA for buprenorphine per year would not be approved.^38^ This PA prohibition in Medicare was associated with a significant increase in per-plan, per-year use of buprenorphine prescriptions.^26^ Notably, because these studies of Medicaid and Medicare used aggregated data, their findings cannot provide information on the extent to which changes in buprenorphine prescription use, if any, were attributable to shifts in treatment retention or initiation. Our study contributes to the literature by using individual-level data to better understand the association between PA prohibitions and treatment retention.

The heterogeneous findings on the association between PA prohibition and buprenorphine prescription use across insurers may be partly explained by variation in plan compliance. Medicare Part D plans are required to report PA requirements to CMS and make these data publicly available, showing high compliance with the CMS mandate to remove PA for buprenorphine.^38^ However, since PA information in private insurance is not readily accessible,^39^ the extent to which private insurance plans complied with PA prohibition laws is unclear. A recent study showed that the percentage of private insurance formularies covering at least 1 buprenorphine product without PA increased from 2017 to 2021 but noted substantial variations across formulations and generic and branded products.^37^ During the same period, the number of states with PA prohibition for any MOUD increased from 7 to 19, including 8 states that fully prohibited PA for all buprenorphine products regardless of formulations or generic or branded status.^23^ Nevertheless, we found no association between PA prohibitions and buprenorphine treatment retention among patients starting treatment with either branded or generic products. Although a new federal regulation will start requiring public reporting of insurance plans’ PA policies in 2027, these rules do not apply to prescription drug PA or most private insurance plans.^40^ This lack of transparency may hinder states’ ability to monitor plan compliance and enforce PA prohibitions effectively.

Another possible mechanism to explain the lack of association between PA prohibitions and 180-day treatment retention is that the impact of PA may be less pronounced for subsequent PAs after treatment has been initiated. A mixed-methods study found that PA was the most commonly cited barrier to prescribing buprenorphine among MOUD prescribers.^41^ Prescribers may prefer buprenorphine products that do not require PA when possible. For patients taking buprenorphine requiring PA, prescribers may become more proficient at navigating the PA process after securing the initial approval. These prescribers may be better prepared to manage subsequent PAs to minimize disruptions in ongoing treatment and reduce the impact of PA on treatment retention among their patients.

In addition to PA, insurance plans can impose other utilization management strategies that can affect buprenorphine treatment retention. These approaches include limits on dosage, quantity, and/or treatment duration; drug testing requirements; step therapy; and mandated concurrent psychosocial counseling.^42,43,44^ Documents from some pharmacy benefit manager companies serving privately insured populations indicate the use of quantity and treatment duration limits for buprenorphine (commonly set at 30 or 90 days).^45,46^ Prior authorization is required once patients reach these limits. This policy structure may partly explain our findings that PA prohibitions were associated with increased short-term (60-day) retention but not longer-term (180-day) retention, as patients are likely to encounter the first PA requirement early in treatment. However, even when PA is prohibited, patients may continue to face barriers to long-term retention due to other utilization management strategies. For example, some states had laws regulating the minimum frequency of required counseling for patients receiving buprenorphine treatment.^47^

Taken together, our findings suggest that adopting PA prohibitions alone may not be associated with improvement in 180-day buprenorphine treatment retention, highlighting the need for additional research and policies to address the gaps in MOUD retention. One potential avenue is for states to strengthen the monitoring and enforcement of existing PA prohibitions. The American Medical Association has actively supported efforts to increase transparency of PA requirements by providing states with resources, such as model legislation and legislative analysis.^48^ These efforts have contributed to the successful passage of PA reform laws in several states.^49,50^ Another approach is to regulate the use of other utilization management strategies. For example, many states have prohibited contracted Medicaid managed care plans from imposing requirements for drug testing, step therapy, and psychosocial therapy for buprenorphine treatment.^42^ A similar approach can be adopted through legislative efforts to cover private insurance.

### Limitations

This study has some limitations. First, we did not have data on plans’ PA requirements, precluding us from verifying whether patients’ treatment was subject to PA. Second, our measure of buprenorphine retention was based on prescriptions filled at retail pharmacies. Patients who received medications from other settings or injectable buprenorphine were not observed, although we expect this population to be small and not likely to significantly affect our findings. Similarly, claims data do not specify the conditions for which buprenorphine was prescribed. Although we have excluded formulations approved exclusively for pain management, we could not determine whether the prescription was intended for OUD. Third, states may have adopted other policies alongside PA prohibitions that could have affected buprenorphine retention. We did not account for the potential impact of such policies, if any. Lastly, we examined the association between implementing PA prohibitions that applied to any buprenorphine product and treatment retention. Because PA prohibitions can affect other outcomes (eg, OUD treatment initiation) and because the scope of PA prohibitions varies across states,^23,51^ future studies can examine other outcomes and the heterogeneous effects of PA prohibitions.

## Conclusions

In this cross-sectional study using a DID approach, we found that the adoption of state laws barring PA in private insurance was not associated with significant changes in 180-day buprenorphine treatment retention. Although many states have adopted PA prohibitions as a strategy to address gaps in OUD treatment, our findings suggest that relying on PA prohibition alone may be insufficient. Additional interventions should be implemented in tandem with PA prohibitions to improve buprenorphine treatment retention.

## Notes

Supplement 1. **eAppendix 1.** States adopting prior authorization prohibition for buprenorphine in private insurance, 2015-2022 **eAppendix 2.** Sample derivation flow chart **eAppendix 3.** Methods of constructing buprenorphine treatment episodes **eAppendix 4.** Assessing the parallel trends assumption in the Callaway and Sant’Anna difference-in-differences (CSDID) method **eAppendix 5.** Results of Callaway and Sant’Anna difference-in-differences (CSDID) models **eAppendix 6.** Results of two-way fixed effect (TWFE) difference-in-differences models **eAppendix 7.** Results of Callaway and Sant’Anna difference-in-differences (CSDID) models assessing 30-day and 60-day retention

Supplement 2.
Data Sharing Statement

## References

[1] Centers for Disease Control and Prevention. Understanding the opioid overdose epidemic. Accessed September 29, 2025. https://www.cdc.gov/overdose-prevention/about/understanding-the-opioid-overdose-epidemic.html

[2] Jones CM, Han B, Baldwin GT, Einstein EB, Compton WM. Use of medication for opioid use disorder among adults with past-year opioid use disorder in the US, 2021. JAMA Netw Open. 2023;6(8):e2327488. doi:10.1001/jamanetworkopen.2023.27488 37548979 PMC10407686

[3] Krawczyk N, Rivera BD, Jent V, Keyes KM, Jones CM, Cerdá M. Has the treatment gap for opioid use disorder narrowed in the U.S.?: a yearly assessment from 2010 to 2019. Int J Drug Policy. 2022;110:103786. doi:10.1016/j.drugpo.2022.103786 35934583 PMC10976290

[4] Shulman M, Wai JM, Nunes EV. Buprenorphine treatment for opioid use disorder: an overview. CNS Drugs. 2019;33(6):567-580. doi:10.1007/s40263-019-00637-z 31062259 PMC6585403

[5] Wakeman SE, Larochelle MR, Ameli O, . Comparative effectiveness of different treatment pathways for opioid use disorder. JAMA Netw Open. 2020;3(2):e1920622. doi:10.1001/jamanetworkopen.2019.20622 32022884 PMC11143463

[6] Chua KP, Nguyen TD, Zhang J, Conti RM, Lagisetty P, Bohnert AS. Trends in buprenorphine initiation and retention in the United States, 2016-2022. JAMA. 2023;329(16):1402-1404. doi:10.1001/jama.2023.1207 37097363 PMC10130945

[7] Dong H, Stringfellow EJ, Russell WA, Jalali MS. Racial and ethnic disparities in buprenorphine treatment duration in the US. JAMA Psychiatry. 2023;80(1):93-95. doi:10.1001/jamapsychiatry.2022.3673 36350592 PMC9647560

[8] Centers for Medicare & Medicaid Services. Quality ID #468: continuity of pharmacotherapy for opioid use disorder (OUD). Accessed January 16, 2025. https://qpp.cms.gov/docs/QPP_quality_measure_specifications/CQM-Measures/2024_Measure_468_MIPSCQM.pdf

[9] Bentzley BS, Barth KS, Back SE, Book SW. Discontinuation of buprenorphine maintenance therapy: perspectives and outcomes. J Subst Abuse Treat. 2015;52:48-57. doi:10.1016/j.jsat.2014.12.011 25601365 PMC4382404

[10] Bell J, Trinh L, Butler B, Randall D, Rubin G. Comparing retention in treatment and mortality in people after initial entry to methadone and buprenorphine treatment. Addiction. 2009;104(7):1193-1200. doi:10.1111/j.1360-0443.2009.02627.x 19563562

[11] Fiellin DA, Schottenfeld RS, Cutter CJ, Moore BA, Barry DT, O’Connor PG. Primary care-based buprenorphine taper vs maintenance therapy for prescription opioid dependence: a randomized clinical trial. JAMA Intern Med. 2014;174(12):1947-1954. doi:10.1001/jamainternmed.2014.5302 25330017 PMC6167926

[12] Degenhardt L, Bucello C, Mathers B, . Mortality among regular or dependent users of heroin and other opioids: a systematic review and meta-analysis of cohort studies. Addiction. 2011;106(1):32-51. doi:10.1111/j.1360-0443.2010.03140.x 21054613

[13] Berg S. What doctors wish patients knew about prior authorization. American Medical Association. Accessed October 31, 2025. https://www.ama-assn.org/practice-management/prior-authorization/what-doctors-wish-patients-knew-about-prior-authorization

[14] Lanham HJ, Papac J, Olmos DI, . Survey of barriers and facilitators to prescribing buprenorphine and clinician perceptions on the Drug Addiction Treatment Act of 2000 waiver. JAMA Netw Open. 2022;5(5):e2212419. doi:10.1001/jamanetworkopen.2022.12419 35552721 PMC9099423

[15] Kermack A, Flannery M, Tofighi B, McNeely J, Lee JD. Buprenorphine prescribing practice trends and attitudes among New York providers. J Subst Abuse Treat. 2017;74:1-6. doi:10.1016/j.jsat.2016.10.005 28132694

[16] Landis RK, Opper I, Saloner B, . Buprenorphine treatment episode duration, dosage, and concurrent prescribing of benzodiazepines and opioid analgesics: the effects of Medicaid prior authorization policies. Drug Alcohol Depend. 2022;241:109669. doi:10.1016/j.drugalcdep.2022.109669 36332589 PMC10695272

[17] Chino F, Baez A, Elkins IB, Aviki EM, Ghazal LV, Thom B. The patient experience of prior authorization for cancer care. JAMA Netw Open. 2023;6(10):e2338182. doi:10.1001/jamanetworkopen.2023.38182 37851442 PMC10585404

[18] Kyle MA, Keating NL. Prior authorization and association with delayed or discontinued prescription fills. J Clin Oncol. 2024;42(8):951-960. doi:10.1200/JCO.23.01693 38086013 PMC10927330

[19] American Medical Association. 2024 AMA prior authorization physician survey. Accessed September 29, 2025. https://www.ama-assn.org/system/files/prior-authorization-survey.pdf

[20] Substance Abuse and Mental Health Services Administration. 2023 National Survey on Drug Use and Health (NSDUH). https://www.samhsa.gov/data/data-we-collect/nsduh-national-survey-drug-use-and-health/datafiles

[21] PAN Foundation. Patient experience with prior authorization. Accessed January 16, 2025. https://www.panfoundation.org/wp-content/uploads/2024/05/Prior-authorization-polling-results-May-2024.pdf

[22] Pollitz K, Pestaina K, Lopes L, Wallace R, Lo J. Consumer survey highlights problems with denied health insurance claims. Kaiser Family Foundation. Accessed January 16, 2025. https://www.kff.org/affordable-care-act/issue-brief/consumer-survey-highlights-problems-with-denied-health-insurance-claims/

[23] Hu JC, Hutchings K, Jalali A, Kapadia SN, Bao Y, Underhill K. State laws banning prior authorization for medications for opioid use disorder increased substantially, 2015-23. Health Aff (Millwood). 2025;44(11):1369-1377. doi:10.1377/hlthaff.2025.00191 41183241 PMC12721913

[24] Christine PJ, Larochelle MR, Lin LA, McBride J, Tipirneni R. Removal of Medicaid prior authorization requirements and buprenorphine treatment for opioid use disorder. JAMA Health Forum. 2023;4(10):e233549. doi:10.1001/jamahealthforum.2023.3549 37862034 PMC10589810

[25] Keshwani S, Maguire M, Goodin A, Lo-Ciganic WH, Wilson DL, Hincapie-Castillo JM. Buprenorphine use trends following removal of prior authorization policies for the treatment of opioid use disorder in 2 state Medicaid programs. JAMA Health Forum. 2022;3(6):e221757. doi:10.1001/jamahealthforum.2022.1757 35977240 PMC9233239

[26] Mark TL, Parish WJ, Zarkin GA. Association of formulary prior authorization policies with buprenorphine-naloxone prescriptions and hospital and emergency department use among Medicare beneficiaries. JAMA Netw Open. 2020;3(4):e203132. doi:10.1001/jamanetworkopen.2020.3132 32310285 PMC7171554

[27] Observational health data sciences and informatics. Athena. Accessed September 29, 2025. https://athena.ohdsi.org/search-terms/start

[28] Micromedex RED BOOK. Merative. Accessed September 29, 2025. https://www.merative.com/documents/micromedex-red-book

[29] US Food and Drug Administration. National Drug Code Directory. Accessed September 29, 2025. https://open.fda.gov/apis/drug/ndc/download/

[30] RxNorm files. The National Library of Medicine. Accessed September 29, 2025. https://www.nlm.nih.gov/research/umls/rxnorm/docs/rxnormfiles.html

[31] Identifying beneficiaries with a treated substance use disorder (SUD). Medicaid and CHIP Business Information Solutions. 2021. Accessed September 29, 2025. https://www.medicaid.gov/medicaid/data-and-systems/downloads/macbis/sud_techspecs.docx

[32] Opioid national drug code and oral MME conversion file update. Centers for Disease Control and Prevention National Center for Injury Prevention and Control. February 1, 2023. Accessed July 3, 2023. https://archive.cdc.gov/www_cdc_gov/opioids/data-resources/index_1715780741.html

[33] Meinhofer A, Williams AR, Johnson P, Schackman BR, Bao Y. Prescribing decisions at buprenorphine treatment initiation: do they matter for treatment discontinuation and adverse opioid-related events? J Subst Abuse Treat. 2019;105:37-43. doi:10.1016/j.jsat.2019.07.010 31443889 PMC6731543

[34] Hurley RW, Bland KT, Chaskes MD, Guth D, Hill EL, Adams MCB. Evidence-based framework for identifying opioid use disorder in administrative data: a systematic review and methodological development study. Pain Med. Published online August 25, 2025. doi:10.1093/pm/pnaf116 40854117 PMC12865102

[35] Goodman-Bacon A. Difference-in-differences with variation in treatment timing. J Econom. 2021;225(2):254-277. doi:10.1016/j.jeconom.2021.03.014

[36] Callaway B, Sant’Anna PHC. Difference-in-differences with multiple time periods. J Econom. 2021;225(2):200-230. doi:10.1016/j.jeconom.2020.12.001

[37] Nguyen TD, Chua KP, Andraka-Christou B, Bradford WD, Simon K. Trends in buprenorphine coverage and prior authorization requirements in US commercial formularies, 2017-2021. JAMA Health Forum. 2022;3(7):e221821. doi:10.1001/jamahealthforum.2022.1821 35977219 PMC9270692

[38] Mark TL, Parish W, Zarkin GA. Association between Medicare and FDA policies and prior authorization requirements for buprenorphine products in Medicare part D plans. JAMA. 2019;322(2):166-167. doi:10.1001/jama.2019.6581 31287514 PMC6618771

[39] Mark TL, Parish WJ, Weber EM, Zarkin GA. Prior authorization for opioid use disorder versus pain medications: lessons learned for parity enforcement. J Stud Alcohol Drugs. 2021;82(2):214-218. doi:10.15288/jsad.2021.82.214 33823968

[40] Pestaina K, Lo J, Wallace R, Long M. Final prior authorization rules look to streamline the process, but issues remain. Kaiser Family Foundation. Accessed October 31, 2025. https://www.kff.org/private-insurance/final-prior-authorization-rules-look-to-streamline-the-process-but-issues-remain/

[41] Haffajee RL, Andraka-Christou B, Attermann J, Cupito A, Buche J, Beck AJ. A mixed-method comparison of physician-reported beliefs about and barriers to treatment with medications for opioid use disorder. Subst Abuse Treat Prev Policy. 2020;15(1):69. doi:10.1186/s13011-020-00312-3 32928272 PMC7491096

[42] Peterson LA, Andrews CM, Abraham AJ, Westlake MA, Grogan CM. Most states allow Medicaid managed care plans discretion to restrict substance use disorder treatment benefits. Health Aff (Millwood). 2024;43(7):1038-1046. doi:10.1377/hlthaff.2023.01023 38950296

[43] Shoulders A, Andrews CM, Westlake MA, Abraham AJ, Grogan CM. Changes in Medicaid fee-for-service benefit design for substance use disorder treatment during the opioid crisis, 2014 to 2021. JAMA Health Forum. 2023;4(8):e232502. doi:10.1001/jamahealthforum.2023.2502 37566428 PMC10422193

[44] Report to Congress: utilization management of medication-assisted treatment in Medicaid. Medicaid and CHIP Payment and Access Commission. Accessed September 29, 2025. https://www.macpac.gov/wp-content/uploads/2019/10/Report-to-Congress-Utilization-Management-of-Medication-Assisted-Treatment-in-Medicaid.pdf

[45] CVS Caremark. Quantity limit: post limit prior authorization buprenorphine sublingual tablets. Accessed December 27, 2025. https://info.caremark.com/content/dam/enterprise/caremark/microsites/dig/pdfs/pa_forms_default/2328-HJ_Buprenorphine.pdf

[46] Prime Therapeutics LLC. Buprenorphine, buprenorphine/naloxone for opioid dependence quantity limit program summary. Accessed December 27, 2025. https://web.primetherapeutics.com/provider/external/commercial/common/doc/en-us/NetResults_Clinical_Criteria_Choice_Buprenorphine_Buprenorphine_Naloxone.pdf?utm_source=chatgpt.com

[47] Andraka-Christou B, Gordon AJ, Bouskill K, . Toward a typology of office-based buprenorphine treatment laws: themes from a review of state laws. J Addict Med. 2022;16(2):192-207. doi:10.1097/ADM.0000000000000863 34014209 PMC8599526

[48] Prior authorization reform initiatives. American Medical Association. Accessed January 16, 2025. https://www.ama-assn.org/practice-management/prior-authorization/prior-authorization-reform-initiatives

[49] 2024 Prior authorization (PA) state law chart. American Medical Association. Accessed September 29, 2025. https://www.ama-assn.org/system/files/prior-authorization-state-law-chart.pdf

[50] Henry TA. 10 states have tackled prior authorization so far in 2024. American Medical Association. Accessed September 29, 2025. https://www.ama-assn.org/practice-management/prior-authorization/10-states-have-tackled-prior-authorization-so-far-2024

[51] Andraka-Christou B, Golan O, Totaram R, . Prior authorization restrictions on medications for opioid use disorder: trends in state laws from 2005 to 2019. Ann Med. 2023;55(1):514-520. doi:10.1080/07853890.2023.2171107 36724766 PMC9897778

