# Supplementary material for: State Prior Authorization Prohibitions and Buprenorphine Retention Among Privately Insured Patients
Source: JAMA Health Forum. 2026 Mar 6;7(3):e260012. doi: 10.1001/jamahealthforum.2026.0012 (PMC12966923; doi:10.1001/jamahealthforum.2026.0012)
Supplement: Supplement 1. — eAppendix 1. States adopting prior authorization prohibition for buprenorphine in private insurance, 2015-2022 eAppendix 2. Sample derivation flow chart eAppendix 3. Methods of constructing buprenorphine treatment episodes eAppendix 4. Assessing the parallel trends assumption in the Callaway and Sant’Anna difference-in-differences (CSDID) method eAppendix 5. Results of Callaway and Sant’Anna difference-in-differences (CSDID) models eAppendix 6. Results of two-way fixed effect (TWFE) difference-in-differences models eAppendix 7. Results of Callaway and Sant’Anna difference-in-differences (CSDID) models assessing 30-day and 60-day retention [file jamahealthforum-e260012-s001.pdf]

Supplemental Online Content

Hu J-C, Kapadia SN, Zhang H, Jalali A, Underhill K, Andrews CM, Bao Y. State prior authorization prohibitions and buprenorphine retention among privately insured patients. *JAMA Health Forum*. 2026;7(3):e260012. doi:10.1001/jamahealthforum.2026.0012

eAppendix 1. States adopting prior authorization prohibition for buprenorphine in private insurance, 2015-2022

eAppendix 2. Sample derivation flow chart

eAppendix 3. Methods of constructing buprenorphine treatment episodes

eAppendix 4. Assessing the parallel trends assumption in the Callaway and Sant'Anna difference-in-differences (CSDID) method

eAppendix 5. Results of Callaway and Sant'Anna difference-in-differences (CSDID) models

eAppendix 6. Results of two-way fixed effect (TWFE) difference-in-differences models

eAppendix 7. Results of Callaway and Sant'Anna difference-in-differences (CSDID) models assessing 30-day and 60-day retention

This supplemental material has been provided by the authors to give readers additional information about their work.

© 2026 Hu J-C et al. *JAMA Health Forum*.

eAppendix 1. States adopting prior authorization prohibition for buprenorphine in private insurance, 2015-2022

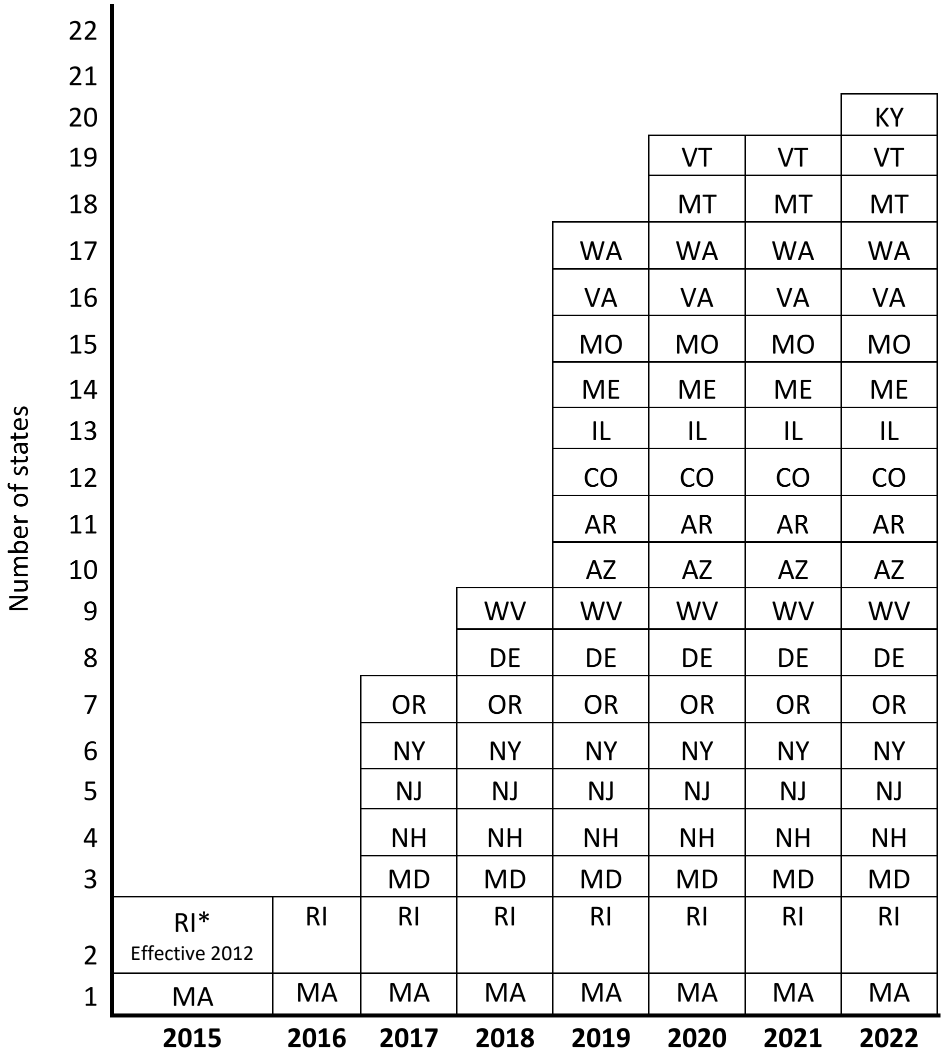

*Prior authorization (PA) prohibition in Rhode Island took effect on 6/18/2012. We excluded Rhode Island from the analysis.

Source: Hu JC, Hutchings K, Jalali A, Kapadia SN, Bao Y, Underhill K. State Laws Banning Prior Authorization For Medications For Opioid Use Disorder Increased Substantially, 2015-23. *Health Aff (Millwood)*. Nov 2025;44(11):1369-1377. doi:10.1377/hlthaff.2025.00191

© 2026 Hu J-C et al. *JAMA Health Forum*.

eAppendix 2. Sample derivation flow chart

Patients aged 18 to 64 who started a new buprenorphine treatment between 1/1/2015 and 6/1/2022

N=206,427

↓

Patients aged 18 to 64 who (1) started a new buprenorphine treatment between 1/1/2015 and 6/1/2022 and (2) met the 6+7 continuous enrollment requirement

N=47,101

↓

Patients aged 18 to 64 who (1) started a new buprenorphine treatment between 1/1/2015 and 6/1/2022, (2) met the 6+7 months continuous enrollment requirement, (3) were not enrolled in an employer self-funded plan in study states.

N=22,946

© 2026 Hu J-C et al. *JAMA Health Forum*.

eAppendix 3. Methods of constructing buprenorphine treatment episodes

We measured the length of each patient's first buprenorphine treatment episode and determined if the length reached 180 days or longer. To construct treatment episodes, we first extracted all pharmacy claims of filled buprenorphine prescriptions. Next, we ordered the claims for each patient by the filled date and used the days of supply to calculate the duration of each prescription. If a patient filled more than one buprenorphine prescription on the same date, we used the maximum days of supply among those prescriptions to calculate the duration of the prescription.

When there was an overlap between prescriptions, we carried the supply of overlapping days forward to the end of the next filled buprenorphine prescription. For example, patient A had two buprenorphine prescriptions: the first was filled on January 1 with a 15-day supply, and the second was filled on January 13 with a 19-day supply. Because there was a 3-day overlap between the two prescriptions, we considered the patient to have 3 more days with buprenorphine in his/her possession. We then recalculated the duration of the second prescription and updated the end date to February 3. Because the patient did not have another buprenorphine prescription filled within 7 days of February 3, we concluded that the treatment episode spanned from January 1 to February 3 (length=34 days).

Example 1 : Patient A

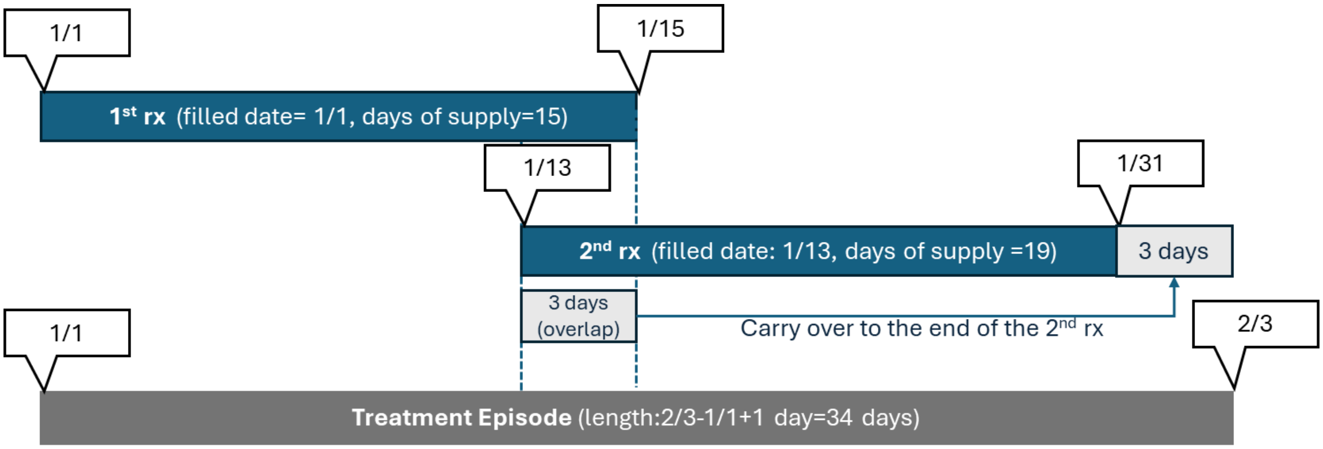

For sensitivity analysis, we allowed different gaps (i.e., 7, 14, and 30 days) between prescriptions. For example, patient B had two buprenorphine prescriptions: the first was filled on January 1 with a 31-day supply, and the second was filled on February 14 with a 10-day supply. When using the CMS standard that only allows a gap of no longer than 7 days between prescriptions, we considered patient B's first treatment episode to span from January 1 to January 31 (length=31 days) because the second prescription was filled more than 7 days after the end of the first prescription. In contrast, when allowing a 14-day or a 30-day gap between prescriptions, we considered patient B's first treatment episode to span from January 1 to February 23 (length=54 days) since the gap between the end of the first prescription and the start of the second prescription did not exceed 14 or 30 days, respectively.

© 2026 Hu J-C et al. *JAMA Health Forum*.

Patient B

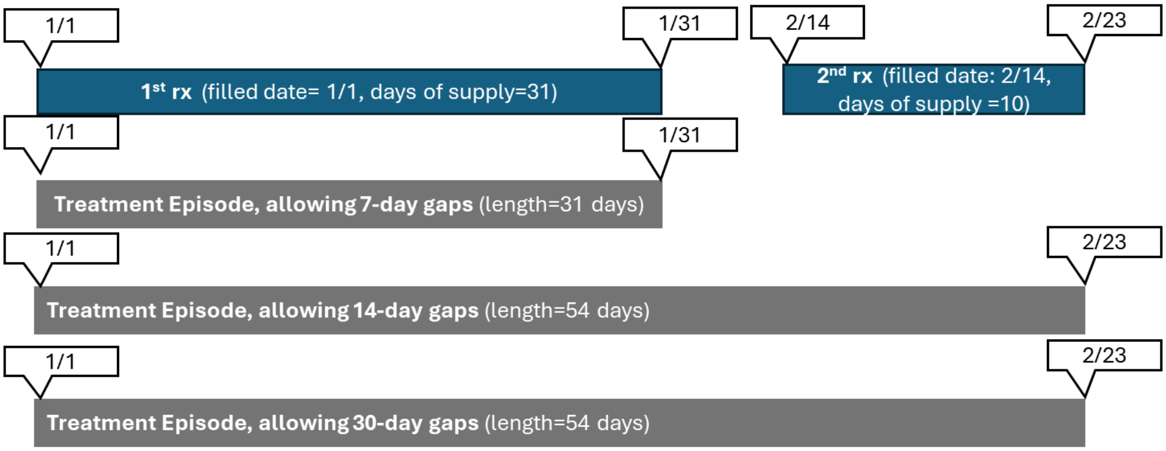

© 2026 Hu J-C et al. *JAMA Health Forum*.

eAppendix 4. Assessing the parallel trends assumption in the Callaway and Sant'Anna difference-in-differences (CSDID) method

The CSDID method provides two ways to assess the parallel trends assumption in a difference-in-differences design.

First, the CSDID method can estimate the mean of average treatment effects (ATT) across all pre-treatment periods (i.e., "pre-treatment average"). In Stata, this estimate can be obtained by using the "*estat event*" post-estimation command. When interpreting the pre-treatment average coefficient, a coefficient that is not significantly different from zero suggests no significant differences in the outcome between the treatment group and the control group during pre-treatment periods.

The CSDID method also supports creating event study plots to visually assess the parallel trends assumption. This can be done by using the "*csdid_plot*" command following the "*estat event*" command. However, these event study plots should not be interpreted in the same way as those from the two-way fixed effects (TWFE) method. This is because, by default, the CSDID method uses different time periods as references when estimating the ATT coefficients during pre-treatment periods (i.e., blue dots in the following figures) and post-treatment periods (red dots in the following figures). Specifically, each pre-treatment coefficient shows the average ATT based on comparisons of consecutive periods (i.e., referencing the previous time period). In contrast, each post-treatment coefficient shows the average ATT based on comparisons relative to the period before treatment (i.e., referencing -1 on the x-axis of the event study plot, similar to the TWFE method).

Please refer to the following sources for more details on the CSDID Stata package and the interpretation of CSDID event study plots:

- Fernando Rios-Avila. Playing with Stata: CSDID Version 1.6. https://friosavila.github.io/playingwithstata/main_csdid.html
- Jonathan Roth. 2024. Interpreting Event-Studies from Recent Difference-in-Differences Methods. https://www.jonathandroth.com/assets/files/HetEventStudies.pdf

We present both the pre-treatment average estimates and the event study plots. Overall, the results suggest no severe violation of the parallel trends assumption.

Using never treated as controls

**Outcome: 180-day retention**	**Pre-treatment average**				
	**Coefficient**	**SE**	**P**	**95% CI**	
allowing 7-day gaps	0.028	0.015	0.067	-0.002	0.059
allowing 14-day gaps	-0.013	0.011	0.232	-0.034	0.008
allowing 30-day gaps	-0.001	0.010	0.899	-0.022	0.019

© 2026 Hu J-C et al. *JAMA Health Forum*.

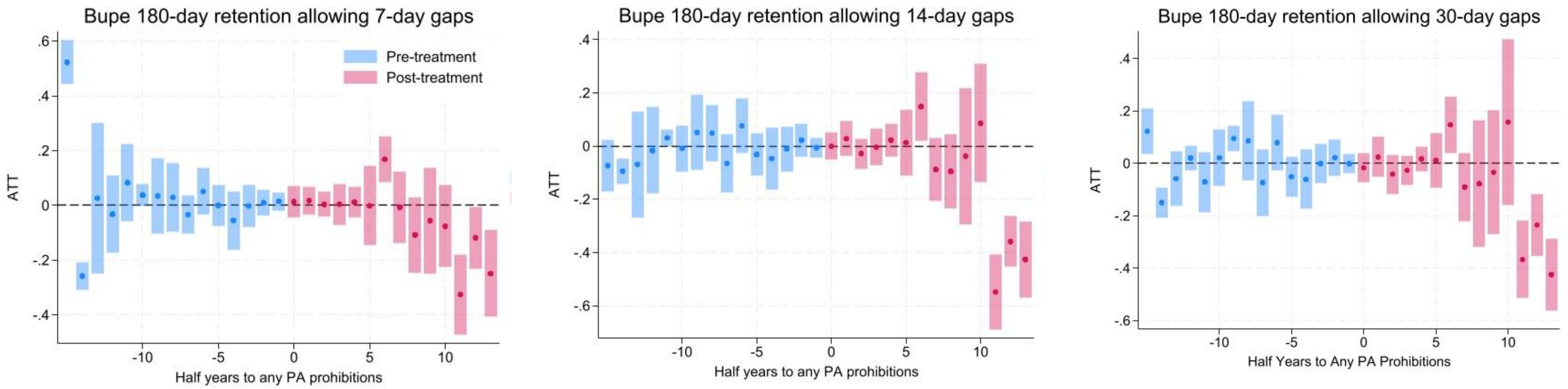

<u>Using not-yet treated as controls</u>

**Outcome: 180-day retention**	**Pre-treatment average**				
	**Coefficient**	**SE**	**P**	**95% CI**	
allowing 7-day gaps	0.029	0.015	1.97	0.049	0.000
allowing 14-day gaps	-0.011	0.011	-1.09	0.276	-0.032
allowing 30-day gaps	0.001	0.010	0.06	0.953	-0.019

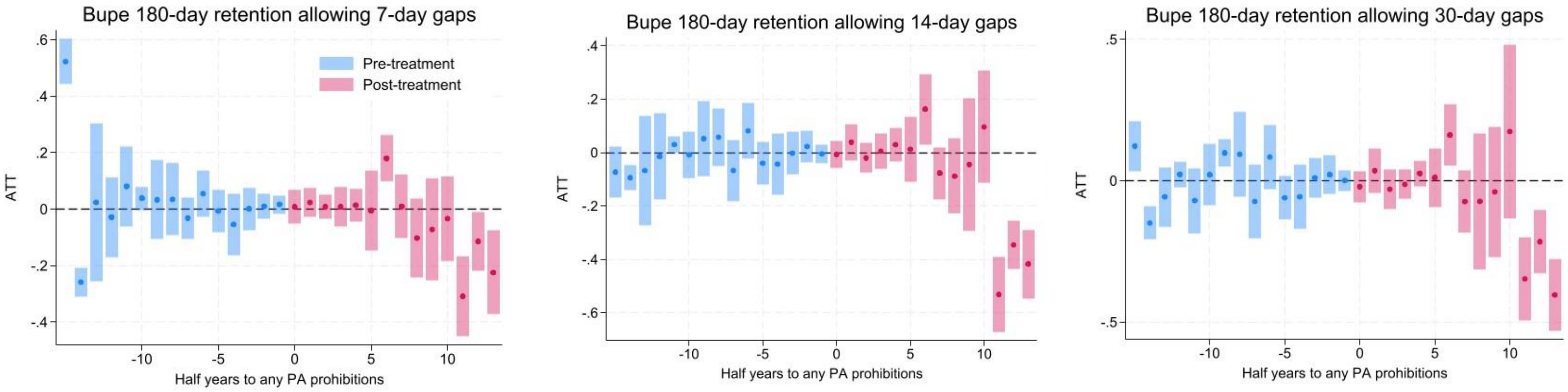

© 2026 Hu J-C et al. *JAMA Health Forum*.

eAppendix 5. Results of Callaway and Sant'Anna difference-in-differences (CSDID) models assessing 180-day retention

Main analysis

CSDID estimate: policy effect of PA prohibition	Using never treated as controls				Using not-yet treated as controls			
**Outcome: 180-day retention**	**estimate**	**95% CI**		**p**	**estimate**	**95% CI**		**p**
allowing 7-day gaps	0.002	-0.050	0.055	0.928	0.007	-0.044	0.059	0.782
allowing 14-day gaps	0.000	-0.067	0.068	0.995	0.006	-0.060	0.072	0.862
allowing 30-day gaps	-0.001	-0.058	0.057	0.98	0.007	-0.048	0.062	0.803

Secondary analysis: Stratified by branded vs generic status of the first buprenorphine prescription

Among patients whose first buprenorphine prescription is branded

CSDID estimate: policy effect of PA prohibition	Using never treated as controls				Using not-yet treated as controls			
**Outcome: 180-day retention**	**estimate**	**95% CI**		**p**	**estimate**	**95% CI**		**p**
allowing 7-day gaps	-0.029	-0.095	0.037	0.39	-0.018	-0.075	0.040	0.553
allowing 14-day gaps	-0.050	-0.107	0.007	0.085	-0.037	-0.090	0.015	0.166
allowing 30-day gaps	-0.037	-0.092	0.018	0.185	-0.028	-0.079	0.023	0.283

Among patients whose first buprenorphine prescription is generic

CSDID estimate: policy effect of PA prohibition	Using never treated as controls				Using not-yet treated as controls			
**Outcome: 180-day retention**	**estimate**	**95% CI**		**p**	**estimate**	**95% CI**		**p**
allowing 7-day gaps	0.040	-0.041	0.120	0.335	0.041	-0.036	0.118	0.297
allowing 14-day gaps	-0.001	-0.086	0.083	0.979	0.003	-0.079	0.084	0.949
allowing 30-day gaps	-0.005	-0.087	0.076	0.904	0.003	-0.075	0.081	0.936

© 2026 Hu J-C et al. *JAMA Health Forum*.

eAppendix 6. Results of two-way fixed effect (TWFE) difference-in-differences models assessing 180-day retention

Main analysis

	No PA prohibition			Any PA prohibition			TWFE estimate: policy effect of PA prohibition			
**Outcome: 180-day retention**	**estimate**	**95% CI**		**estimate**	**95% CI**		**estimate**	**95% CI**		**p**
allowing 7-day gaps	0.296	0.288	0.305	0.322	0.291	0.353	0.026	-0.014	0.065	0.199
allowing 14-day gaps	0.369	0.360	0.378	0.387	0.355	0.419	0.018	-0.023	0.059	0.380
allowing 30-day gaps	0.450	0.441	0.459	0.464	0.431	0.497	0.014	-0.028	0.057	0.516

Secondary analysis: Stratified by branded vs generic status of the first buprenorphine prescription

Among patients whose first buprenorphine prescription is branded

	No PA prohibition			Any PA prohibition			TWFE estimate: policy effect of PA prohibition			
**Outcome: 180-day retention**	**estimate**	**95% CI**		**estimate**	**95% CI**		**estimate**	**95% CI**		**p**
allowing 7-day gaps	0.307	0.303	0.312	0.364	0.330	0.398	0.057	0.018	0.096	0.004*
allowing 14-day gaps	0.384	0.378	0.389	0.432	0.394	0.470	0.048	0.005	0.091	0.028*
allowing 30-day gaps	0.470	0.463	0.476	0.517	0.473	0.560	0.047	-0.003	0.097	0.068

Among patients whose first buprenorphine prescription is generic

	No PA prohibition			Any PA prohibition			TWFE estimate: policy effect of PA prohibition			
**Outcome: 180-day retention**	**estimate**	**95% CI**		**estimate**	**95% CI**		**estimate**	**95% CI**		p
allowing 7-day gaps	0.292	0.276	0.307	0.291	0.256	0.327	0.000	-0.051	0.050	0.997
allowing 14-day gaps	0.361	0.347	0.375	0.353	0.321	0.385	-0.008	-0.054	0.037	0.722
allowing 30-day gaps	0.439	0.425	0.453	0.421	0.389	0.453	-0.018	-0.064	0.027	0.428

Note: * denotes p<0.05. Estimates should be interpreted as the predicted probability of 180-day retention.

© 2026 Hu J-C et al. *JAMA Health Forum*.

eAppendix 7. Results of Callaway and Sant'Anna difference-in-differences (CSDID) models assessing 30-day and 60-day retention

	**CSDID policy effect, using never treated as controls**		**CSDID policy effect, using not-yet treated as controls**	
	Estimate (95% CI)	p	Estimate (95% CI)	p
Outcome: 30-day retention				
Allowing 7-day gaps	0.06 (-0.044, 0.057)	0.802	0.011 (-0.039, 0.060)	0.666
Allowing 14-day gaps	0.012 (-0.042, 0.066)	0.658	0.021 (-0.031, 0.073)	0.434
Outcome: 60-day retention				
Allowing 7-day gaps	0.048 (-0.006, 0.102)	0.084	0.050 (-0.003, 0.104)	0.063
Allowing 14-day gaps	0.050 (-0.003, 0.103)	0.062	0.054 (0.004, 0.105)	0.035*

Note: * denotes p<0.05.

© 2026 Hu J-C et al. *JAMA Health Forum*.
