# Supplementary material for: State Prior Authorization Prohibitions and Buprenorphine Retention Among Privately Insured Patients
Source: JAMA Health Forum. 2026 Mar 6;7(3):e260012. doi: 10.1001/jamahealthforum.2026.0012 (PMC12966923; doi:10.1001/jamahealthforum.2026.0012)
Supplement: Supplement 2. — Data Sharing Statement [file jamahealthforum-e260012-s002.pdf]

Data Sharing Statement

Hu. State Prior Authorization Prohibitions and Buprenorphine Retention Among Privately Insured Patients. *JAMA Health Forum*. Published March 06, 2026.
doi:10.1001/jamahealthforum.2026.0012

Data
Data available: No

Additional Information
Explanation for why data not available: The database used in this study is owned and managed by the Health Care Cost Institute. Information on how to obtain these data is available here: https://healthcostinstitute.org/data-access-hub
